# Spontaneously Piezo- and Pyroelectric Polyacrylonitrile Brushes Prepared via Surface-Initiated Polymerization

**DOI:** 10.1021/jacs.6c10834

**Published:** 2026-08-28

**Authors:** Fei Hu, Dae-Sung Park, Suqiu Jiang, Javier Herrero-Martin, Konstantin Romanyuk, Andrei Kholkin, Dragan Damjanovic, Harm-Anton Klok

**Affiliations:** † Institut Des Matériaux and Institut Des Sciences Et Ingénierie Chimiques, Laboratoire Des Polymères, 27218 École Polytechnique Fédérale de Lausanne (EPFL), Station 12 CH-1015 Lausanne, Switzerland; ‡ Group for Ferroelectrics and Functional Oxides, École Polytechnique Fédérale de Lausanne (EPFL), Station 12, CH-1015 Lausanne, Switzerland; § ALBA Synchrotron, Carrer de la Llum 2-26, Cerdanyola Del Vallès 08290, Spain; ∥ Department of Physics, 56062 CICECO − Aveiro Institute of Materials, University of Aveiro, 3810-193 Aveiro, Portugal; ⊥ Institute of Solid State Physics, University of Latvia, LV-1063 Riga, Latvia

## Abstract

Surface-initiated polymerizations generate thin films (“brushes”) consisting of polymer chains that are anchored with one chain end to a solid surface. As they are conducted from initiator-modified solid substrates, these polymerizations allow for unidirectional chain growth and enforce a stretched chain conformation. Using acrylonitrile as an example of a monomer with a dipolar side-chain functional group, this study finds that surface-initiated polymerization not only impacts the alignment and conformation of the polymer main chain but can also influence the orientation of side-chain functional groups. Surface-initiated polymerization of acrylonitrile is found to result in polyacrylonitrile (PAN) brushes that display spontaneous pyro- and piezoelectric behavior. As these properties are not observed in spin-cast PAN films, this indicates that surface-initiated growth of PAN enforces an overall parallel orientation of the dipolar nitrile side-chain functional groups. This is supported by polarized FTIR spectroscopy and NEXAFS experiments, which suggest an overall nonisotropic orientation of nitrile groups in the PAN brush, whereas the nitrile groups in the spin-cast PAN film are more isotropically oriented. Pyro- and piezoelectric polymers are attractive for use in sensors, actuators, and energy-harvesting devices but typically require electrical poling, mechanical stretching, or electrospinning to promote the alignment of molecular dipoles and enhance piezo- and pyroelectric properties. The ability to prepare thin polymer films that display spontaneous pyro- and piezoelectric behavior is significant as it renders these postprocessing steps unnecessary.

## Graphical abstract

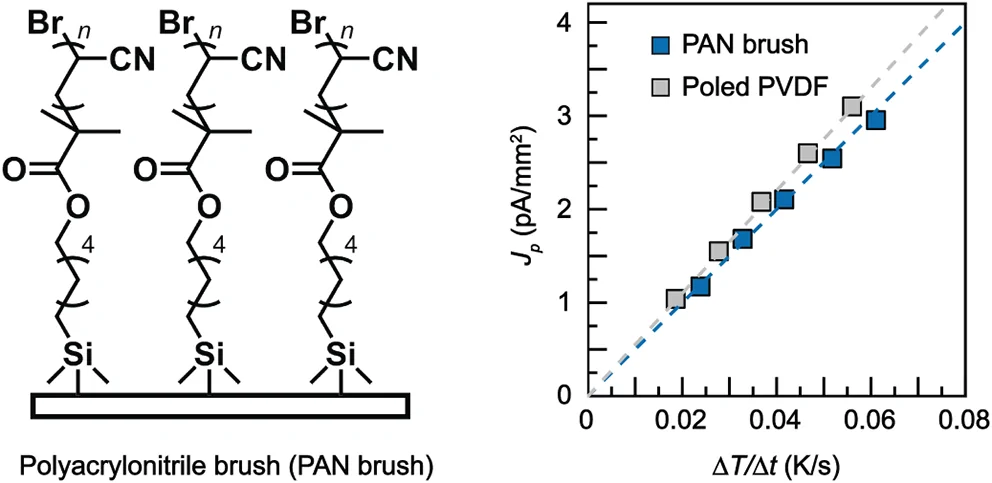

## Introduction

Piezo- and pyroelectric polymers are attractive materials for use in sensors, actuators and energy harvesting devices.^1–6^ In contrast to inorganic piezo- and pyroelectric materials, polymers can be processed at low temperatures, have a low density and possess mechanical properties that make them attractive for the fabrication of flexible devices. Polymers that possess piezo- and pyroelectric properties include biological polymers such as various proteins and cellulose,^7,8^ as well as synthetic polymers.^9–11^ The most prominent synthetic piezo- and pyroelectric polymer is polyvinylidene fluoride (PVDF).^12–16^ Other examples of synthetic piezo- and pyroelectric polymers are polyacrylonitrile (PAN)^17^ and several polyamides.^18^

Whereas inorganic piezo- and pyroelectric materials typically are crystalline solids with a noncentrosymmetric crystal structure,^19,20^ polymers generally are semicrystalline materials with a complex morphology consisting of amorphous and crystalline domains. For polymers to possess pyro- and piezoelectric properties, some degree of polar order, i.e., a net noncentrosymmetric organization of molecular dipoles is required. Prior to use, piezo- and pyroelectric polymers are typically subjected to an additional processing step, such as electrical poling, mechanical stretching or electrospinning to promote the alignment of molecular dipoles and enhance piezo- and pyroelectric properties. Synthetic approaches that allow the spontaneous alignment of molecular dipoles without the need for these additional processing steps would therefore represent a valuable addition to the current strategies for the production of piezo- and pyroelectric polymers.

Surface-initiated controlled radical polymerization allows to produce thin films (also referred to as polymer brushes), which are composed of polymer chains that are tethered with one chain end to a solid surface.^21–26^ Since polymers are grafted from initiator-modified solid substrates, this process allows for unidirectional chain growth and enforces a stretched chain conformation. Surface-initiated polymerization, however, does not only impact alignment and conformation of the main chain of surface-tethered polymers, but can also influence the orientation of side chain functional groups. Second-harmonic generation studies on the surface-initiated polymerization of a chromophore-modified methacrylate, for example, indicated that with increasing polymerization time, the conformation of the polymer changes from a “pancake” conformation to a more stretched conformation, and was accompanied by a change in the tilt angle and orientation of the side chain functional groups.^27^ Other work on side chain liquid crystalline polymer brushes has shown that at high grafting densities the liquid crystalline functional groups align parallel to the surface,^28–30^ whereas at lower grafting densities homeotropic, or a tilted alignment has been observed.^31–33^

This work demonstrates that surface-initiated atom transfer radical polymerization of acrylonitrile, as an example of a monomer with a dipolar side chain functional group, results in thin polymer films that exhibit spontaneous piezo- and pyroelectricity without the need for additional processing steps such as e.g., electric field poling. These findings indicate that surface-initiated polymerization of dipolar side chain functional monomers enforces a net polar side chain orientational order, paving the way for alternative fabrication pathways toward piezo- and pyroelectric polymer thin films.

## Experimental Section

### Materials

All chemicals were used as received unless described otherwise. Basic aluminum oxide, acrylonitrile, polyacrylonitrile (average *M*_v_ = 150,000 g/mol) and 3,7-di­(4-biphenyl)­1-naphthalene-10-phenoxazine (≥97%) were purchased from Sigma-Aldrich. Dimethylacetamide (DMAc) (99%, anhydrous), ethyl α-bromoisobutyrate (95%) and dimethylformamide (DMF) were purchased from Fluorochem. Prior to polymerization, acrylonitrile was passed over an aluminum oxide column to remove the inhibitor. Methanol (analytical grade) was purchased from Fisher Chemical. The ATRP initiator (6-(2-(2-bromo-2-methyl)­propionyloxy)­hexyldimethylchlorosilane) was synthesized following a published procedure.^34^ Deionized water was obtained from a Millipore Direct-Q 5 ultrapure water system. Conductive *p*-type, boron-doped silicon (Si) wafers with a diameter of 100 mm ([100]-crystallographic orientation) were purchased from Sil’ Tronix Silicon Technologies and diced into 10 × 8 mm^2^ pieces. A poled poly­(vinylidene fluoride) (PVDF) film (thickness ≈50 μm) coated with a silver electrode was purchased from Entran (now TE Connectivity), and cut into 8 × 10 mm^2^ rectangular specimens.

### Methods

#### Diffusion-Ordered NMR Spectroscopy (DOSY)

DOSY experiments were run on a Bruker 400 MHz liquid NMR spectrometer at 298 K. The gradient ramp varies linearly from 2.0 to 98.0, and 16 scans per increment were used to construct the decay function. DMSO-d_6_ was used as the solvent. DOSY analysis of the solution polymerized PAN afforded a diffusion coefficient (*D*) of 9.12 × 10^–7^ cm^2^/s. Using this diffusion coefficient, the PAN molecular weight (*M*_wt_) was estimated using log­(η*D*) = −0.3052 log­([η]*M*_wt_) – 8.541264,^35^ where η is the bulk viscosity of the solvent (2.00 mPa·s). This analysis provides a PAN molecular weight of 4200 g/mol. Using σ = (*h*ρ*N*_A_)/*M*_wt_ (nm^–2^),^26^ where σ is the grafting density, *h* is the dry thickness of the polymer brush (5.8 nm, measured by ellipsometry), ρ the bulk density of the polymer (1.18 g/mL), and *N*_A_ Avogadro’s number, the grafting density of the PAN brush is estimated to 0.98 chains/nm^2^.

#### Water Contact Angle Analysis

Water contact angle measurements were performed at ambient conditions using a DataPhysics OCA 35 contact angle measurement instrument. A 15 μL Milli-Q water droplet was used for each measurement. The contact angle was determined using the software SCA20 from DataPhysics. The reported values are given as average value of at least 3 measurements ± standard deviation.

#### X-ray Photoelectron Spectroscopy (XPS)

XPS measurements were carried out on a VersaProbe II instrument (Physical Electronics Inc.) using the monochromated Kα X-ray line of an aluminum anode. The pass energy was set to 46.95 eV with a step size of 0.2 eV. The samples were electrically isolated from the sample holder and charges were compensated. The spectra were referenced at 284.8 eV using the C–C bound component of the C 1s transition.

#### Ellipsometry

Dry film thicknesses (*h*) were measured using a SemiLAB SE2000 instrument at an incident angle of 70°. The film thickness was fitted using a four-layer silicon/silicon oxide/polymer brush/air model. The refractive index n was given by the Cauchy approximation,

$$n\left(\lambda \right)=A+\frac{B}{\lambda^{2}}+\frac{C}{\lambda^{4}}+...$$

where λ represents the wavelength of the incident light. *A*, *B* and *C* are material-dependent empirical coefficients. For the fitting of the polyacrylonitrile brushes a refractive index of 1.51 was used.^36^ Measurements were taken from three different points for each substrate. Dry film thicknesses are reported as the average ± standard deviation (SD) of these three values. Topographical maps were obtained by measuring the intersection points of 5 × 5 rows per wafer. The ellipsometry data was analyzed with the SAM suite software from SemiLAB.

#### Atomic Force Microscopy (AFM)

Atomic force microscopy experiments were conducted using a Cypher VRS instrument (Asylum Research) operated in tapping mode. Al-coated silicon cantilevers (HQ:NSC14/Al BS, MikroMasch) with a nominal spring constant of 5.0 N·m^–1^ were employed. The topographic image data was acquired at room temperature under ambient conditions, and subsequently processed and analyzed using the open-source software Gwyddion.

#### Piezoresponse Force Microscopy (PFM)

Single-frequency out-of-plane PFM measurements were performed using a Cypher VRS atomic force microscope (Asylum Research) and a Ntegra Aura (NT-MDT) instrument (for the results presented in Supporting Information Figure S7) using Pt-coated conductive probes (HQ:NSC18/Pt, MikroMasch) with a nominal spring constant of 2.8 N·m^–1^ and a resonance frequency of ≈75 kHz. The measurements were conducted at room temperature under ambient conditions with an AC excitation voltage (*V*_AC_) ranging from 1–5 V at a frequency of approximately 360 kHz. Low-frequency response was used for imaging. To determine effective out-of-plane piezoelectric coefficients (*d*_33_), *V*_AC_ with amplitude ranging from 1–5 V was applied to the tip, and the resulting cantilever oscillation was detected via optical lever deflection and recorded as a photodetector voltage signal. To convert the photodetector output to mechanical displacement, an empirical optical lever sensitivity *S* in the range of 40–80 nm/V was used. This range was based on previous calibrations of the instrument and prior experience with similar cantilevers and sample systems. The dynamic response was corrected by dividing the signal by the quality factor *Q*, which was determined for each measurement from contact resonance frequency sweeps and ranged between 100 and 300 across all data. The corrected displacement amplitude Δ*x* was then normalized by the applied *V*_AC_ to yield an approximate effective *d*_33_:

$$d_{33}\approx \frac{A_{\mathrm{raw}}}{S\cdot Q\cdot V_{\mathrm{AC}}}$$

where *A*_raw_ is the measured amplitude in photodetector voltage.

#### Polarized Fourier-Transform Infrared (FTIR) Spectroscopy

Measurements were performed using a PerkinElmer Spectrum 3 FTIR spectrometer equipped with a Specac grid polarizer (ZnSe). For these experiments, polymer brushes were grafted from glass substrates instead of silicon wafers. The spectrometer was operated in transmission mode. Spectra were acquired at room temperature over the range of 3600–1400 cm^–1^ with a spectral resolution of 4 cm^–1^. Spectra were obtained by recording 32 scans for the PAN brush sample, and 64 scans for the spin-coated PAN film. Polarized infrared light was applied in two orthogonal orientations: 0° (parallel) and 90° (perpendicular). For each polarization angle, background spectra were recorded. The spectra underwent baseline correction, and were normalized with respect to the intensity of the CH_2_/CH stretch vibration at ∼2921 cm^–1^.

#### Near Edge X-ray Absorption Fine Structure (NEXAFS) Spectroscopy

NEXAFS measurements were performed at beamline BL29-BOREAS^37^ of the ALBA Synchrotron (Barcelona, Spain) in total electron-yield (TEY) detection mode under ultrahigh vacuum conditions (2 × 10^–10^ mbar). The X-ray beam was focused down to ∼110 μm × 80 μm (horizontal × vertical), and an estimated photon flux was reduced to ∼10^10^ s^–1^ at the C K edge. XLD (X-ray Linear Dichroism) spectra were obtained by subtracting normalized XAS spectra recorded using horizontal linearly polarized X-rays from those acquired using vertically polarized X-rays.

#### Dynamic Pyroelectric Current Measurements

The pyroelectric current, *i*_p_, is generated by a time-dependent temperature variation (d*T*/d*t*), and is given by

$$i_{p}=\mu_{p}\frac{dT}{dt}A$$

where μ_p_ is the pyroelectric coefficient of the material, *A* the electrode area over which the current is collected, and d*T*/d*t* the rate of temperature change. Accordingly, the pyroelectric current is linearly proportional to the rate of temperature change, d*T*/d*t*, rather than the time the sample is held at a given temperature (d*T*/d*t* = 0). Dynamic pyroelectric currents (*i*_p_) were measured with a custom-made device that combines an electrometer, an analog proportional integral derivative (PID) temperature controller, and a power supply.^38–41^ Pyroelectric current measurements were performed while applying a triangular or a square temperature waveform with amplitudes of 1–3 K and frequencies of 10 or 20 mHz. Samples were placed on a copper stage with concentric knife edges to avoid secondary effects that may arise in the case of sample clamping. The top surfaces of the samples were electrically contacted with a cantilever probe fixed on a micropositioner (XYZ 300TR, Quarter Research & Development, Oregon, U.S.). During all measurements, samples were enclosed in a PTFE housing.

### Procedures

#### ATRP-Initiator Functionalization of Silicon Wafers

Conductive rectangular silicon wafers (0.8 × 1 cm^2^) were sonicated in methanol, deionized water, and acetone for 5 min, respectively. After drying under a flow of nitrogen, the wafers were cleaned with a Femto O_2_ plasma system for 25 min (200 W, Diener Electronics), and subsequently 12 pieces of wafer were mounted in a custom-made holder (5% carbon-reinforced PTFE) that was placed in a glass reactor. The reactor was evacuated and backfilled with nitrogen three times. Then, a solution of the initiator (0.1 mmol, 35 μL in 10 mL dry toluene) was added to the wafers under nitrogen. After stirring for 48 h at room temperature under N_2_ atmosphere, the wafers were removed from the glass reactor, rinsed with toluene, methanol, deionized water, and acetone, dried using a stream of nitrogen, and stored under vacuum until further use.

#### Photoinduced Surface-Initiated Atom Transfer Radical Polymerization of Acrylonitrile

First 3,7-di­(4-biphenyl)­1-naphthalene-10-phenoxazine (Phenox-O-PC) (4 mg, 6.52 μmol) was added to a round-bottom flask that was equipped with a septum and wrapped in aluminum foil. Then, 5.5 mL acrylonitrile (4.46 g, 0.084 mol) and 1.2 mL DMAc were added, and the solution degassed via three freeze–pump–thaw cycles.

Surface-initiated polymerizations were performed in a glass reactor equipped with a septum on one side and connected to the Schlenk line on the other side (Supporting Information Figure S1). Several drops of DMAc were added on the glass lid of the reactor before a Petri dish was placed on top. The thin DMAc film that forms prevents light scattering between the two glass parts. ATRP initiator functionalized silicon wafers were placed upside down in the Petri dish on top of smaller pieces of bare silicon wafers as spacers. Then, the greased glass reactor was placed on top, and tightly sealed with a metal ring. The apparatus was evacuated and backfilled with nitrogen thrice, and the solution containing the monomer and photocatalyst slowly added using a nitrogen purged syringe. The reactor was placed on a metallic support above a LED source (M405LP1-C1, Thorlabs, monochromatic excitation at 405 nm) and irradiated from the bottom at an intensity of 1.33 mW/cm^2^ (confirmed by a photodiode sensor S170C, Thorlabs, 18 × 18 mm^2^ active sensor area, λ = 350–1100 nm, 10 nW–150 mW) under nitrogen atmosphere. Polymerizations were conducted for 4 h at room temperature. After that, the reactor was opened, and the wafer was placed in a vial containing 2–3 mL DMAc, and shaken for about 10 min. The wafers were rinsed with acetone again then dried using a stream of nitrogen and stored under vacuum.

To estimate the grafting density, a PAN brush was grown following the procedure outlined above, but in the presence of a sacrificial initiator, ethyl α-bromoisobutyrate (1 μL, 6.5 μmol), which was added to the DMAc solution containing the monomer and the photoredox catalyst. The molecular weight of the solution-polymerized PAN was determined by DOSY NMR analysis of the DMAc solution after a period of 4 h.

#### Preparation of Spin-Coated Polyacrylonitrile Films

A 10 mg/mL solution of polyacrylonitrile in DMF was prepared by stirring at room temperature for 24 h. The polymer solution was then spin coated on clean and plasma-treated silicon wafers (1 cm × 0.8 cm^2^) using a Sawatec LSM-250 spin-coater. Spin-coating was performed at 6000 rpm for 40 s, using 30 μL solution to cover the wafers. The samples were then annealed at 70 °C for 30 min, and subsequently dried under vacuum overnight at room temperature.

#### Sample Preparation for Dynamic Pyroelectric Current Measurements

For the analysis of dynamic pyroelectric properties, a top electrode was evaporated on the polyacrylonitrile brush-modified conductive silicon wafers. Aluminum electrodes were deposited using the Alliance-Concept EVA 760 thermal evaporator at the EPFL Center of MicroNanoTechnology (CMi) using a custom stainless-steel shadow mask (5 cm × 5 cm × 1 mm) with 400 square apertures (1 mm^2^ each). The deposition rate, which varied depending on the target thickness, typically ranged from subnanometer per second to several nanometers per second. The final film thickness was preprogrammed and precisely regulated by the system’s integrated quartz crystal microbalance (Inficon IC/6). The thickness of the top electrode is comparable to that of the polymer brush or spin coated film, i.e., ∼10 nm. The size of the pads is determined by the thermal evaporation mask employed, with a mask area of 1 mm^2^ specified in this work. Typically, 10–12 electrode pads were deposited on a single polymer brush sample.

## Results and Discussion

Surface-initiated polymerization experiments in this paper utilized acrylonitrile as a prototypical dipolar side chain functional monomer. The corresponding homopolymer, polyacrylonitrile (PAN) contains nitrile side chain functional groups with a dipole moment of 3.5 D.^42^ First reports on the pyro- ^43,44^ and piezoelectric^42,45^ properties of this polymer date back to the 1970s and 1980s. For the work reported here, polyacrylonitrile brushes were grown from 6-(2-(2-bromo-2-methyl)­propionyloxy)­hexyldimethylchlorosilane-modified silicon substrates via photoinduced, metal free surface-initiated atom transfer radical polymerization (ATRP) using 3,7-di­(4-biphenyl)­1-naphthalene-10-phenoxazine (Phenox-OPC)^46^ as photoredox catalyst (Scheme 1). The polymerizations were conducted at room temperature under nitrogen atmosphere in dimethylacetamide (DMAc) using a 405 nm LED light source.

**Scheme 1:**
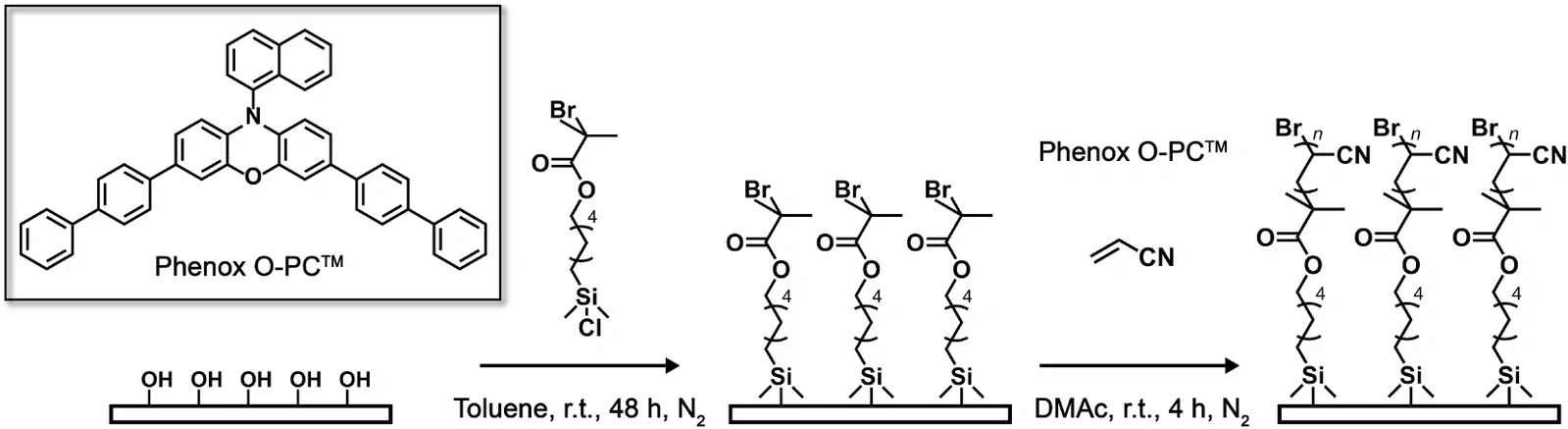
Photoinduced Surface-Initiated Atom Transfer Radical Polymerization of Acrylonitrile from Silicon Substrates

The polyacrylonitrile brushes were characterized with ellipsometry, atomic force microscopy, water contact angle analysis and X-ray photoelectron spectroscopy (XPS). Ellipsometry analysis of PAN brushes obtained after a polymerization time of 4 h indicated a dry film thickness of 13.88 ± 1.11 nm. These results were confirmed by atomic force microscopy analysis of the edge of the PAN brush, which revealed a film thickness of ∼10 nm (Supporting Information Figure S2). Figure 1A presents the static water contact angles of an unmodified, clean silicon wafer, the ATRP initiator-modified substrate and the PAN brush. Modification of the silicon substrate with the ATRP initiator, and subsequent grafting of the PAN brush resulted in a change in the water contact angle from 18.3° to 81.7° and 55.4°. Figure 1B presents the XPS C 1s high resolution XPS scan of the PAN brush (the XPS survey spectrum and the N 1s high resolution scan of the PAN brush are included in Supporting Information Figure S3). The C 1s high resolution spectrum of the PAN brush can be fitted with 3 residuals at binding energies of 286.74, 285.84, and 284.94 eV, corresponding to the CN, CH and CH_2_ carbon atoms of the repeating unit of the polymer in the expected 1:1:1 peak area ratios.^47,48^ To estimate the grafting density of the brushes, surface-initiated atom transfer radical polymerization of acrylonitrile was conducted in the presence of ethyl α-bromoisobutyrate as sacrificial initiator. From the molecular weight of the solution polymerized PAN, which was obtained from DOSY NMR (Supporting Information Figure S4), a grafting density of 0.98 chains/nm^2^ was obtained.

**Figure 1:**
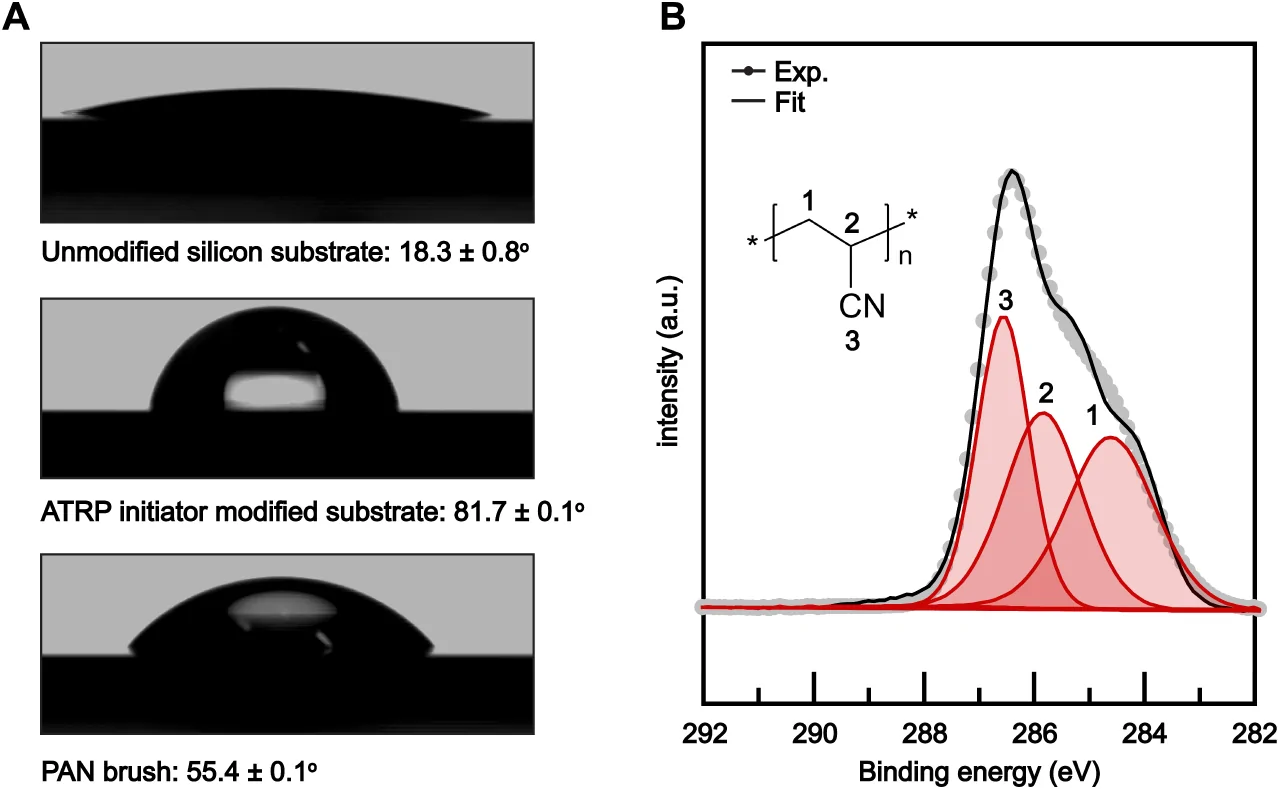
(**A**) Water contact angle analysis of an unmodified silicon substrate, an ATRP initiator modified substrate, and a PAN brush grafted from a silicon substrate. (**B**) C 1s high resolution XPS scan of a PAN brush (*h* = 10 nm) grafted from a silicon substrate.

To investigate the impact of surface-initiated polymerization on the orientation of the dipolar nitrile groups, the pyro- and piezoelectric properties of the polymer thin films were studied. A net parallel alignment of nitrile groups would result in PAN films with polar order that would be expected to exhibit pyro- and piezoelectric behavior. Polymer thin films in which the nitrile groups are isotropically oriented would not be expected to exhibit these properties. To highlight the possible influence of polymer main chain conformation on pyro- and piezoelectric properties, experiments were also conducted on a spin-coated PAN film with a thickness of 10 nm. A commercially available, electrically poled PVDF film (thickness = 50 μm) was used as a further control. To assess their pyroelectric properties, 12–16 1 × 1 mm^2^ aluminum electrodes were deposited on the PAN brushes as an out-of-plane capacitor (Figure 2A). The electrode-modified brushes were subsequently placed on a copper sample stage within the pyroelectric current measurement setup. Pyroelectric current measurements were conducted by applying a triangular or square temperature waveform with amplitudes of Δ*T* = 1–3 K at frequencies of *f* = 10 or 20 mHz, while measuring the resulting electric output current in time.

**Figure 2:**
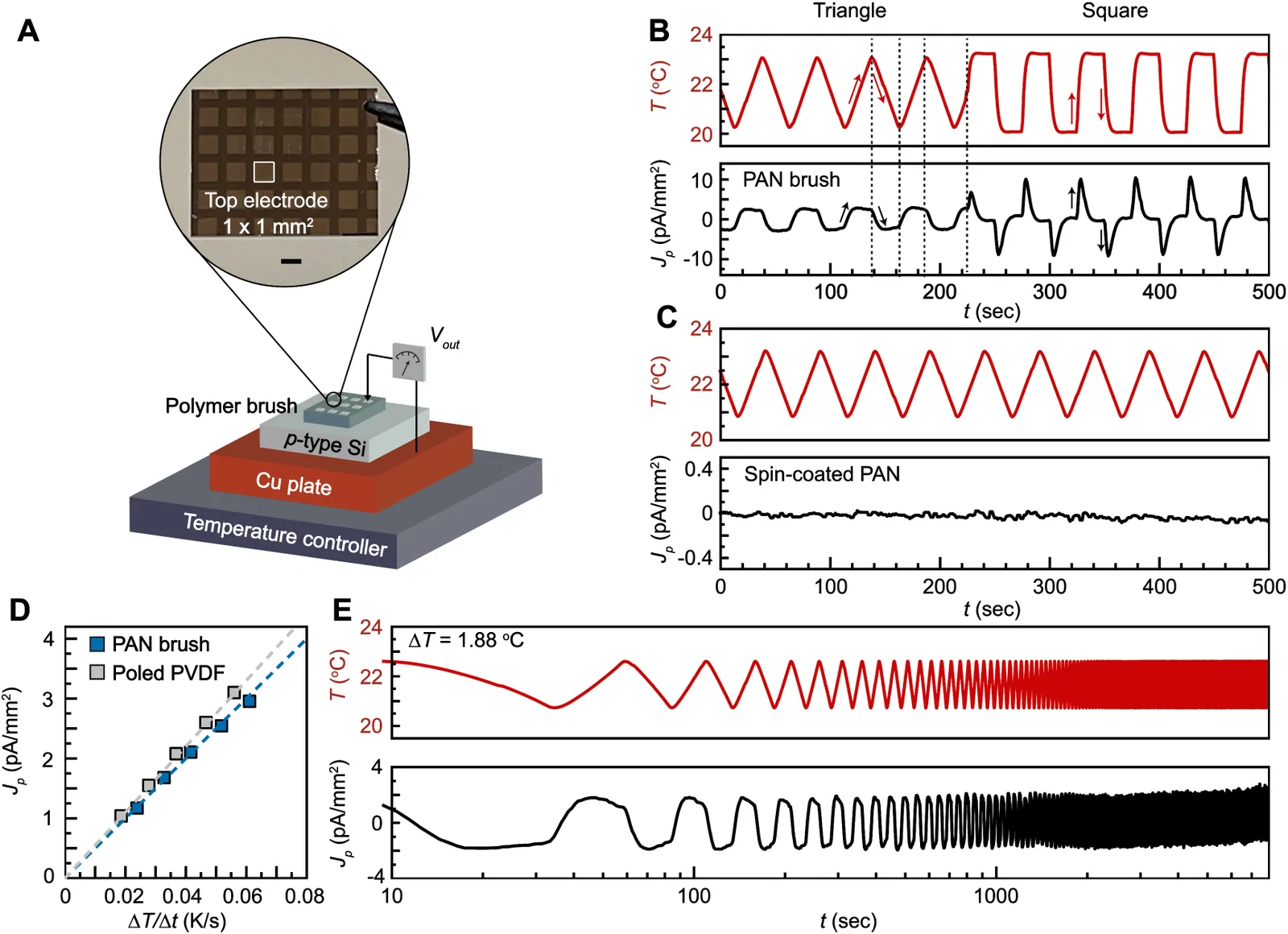
(**A**) Schematic illustration of the experimental setup used for the measurement of the dynamic pyroelectric response of polymer thin films. (**B**) The pyroelectric current density, *J*_p_, of a PAN brush measured by continuously applying triangular and square temperature waveforms in time (*t*) with an amplitude of Δ*T* = 3.0 K at *f* = 20 mHz. (**C**) The *J*_p_ of a 10 nm spin-coated PAN film, measured by applying a triangular temperature waveform with an amplitude of Δ*T* = 2.0 K at *f* = 20 mHz. (**D**) Comparison of the *J*_p_ of the PAN brush and a PVDF film as a function of Δ*T*/Δ*t*. (**E**) *J*_p_ of a PAN brush exposed to a continuous triangular temperature waveform with Δ*T* = 1.88 K at *f* = 20 mHz over a period of 8000 s.

Figure 2B presents the pyroelectric current density (*J*_p_ = *i*_P_/A) that was measured by applying a triangular and square wave temperature modulation of 3 K to a PAN brush. Application of a triangular temperature modulation results in a pyroelectric current density of ∼2.2 pA/mm^2^, whereas a pyroelectric current density of ∼10 pA/mm^2^ was detected when the PAN brush was subjected to a square-wave temperature modulation. The higher pyroelectric current that is detected upon application of the square-wave temperature modulation reflects the increased rate of temperature change (Δ*T*/Δ*t*) under these conditions, as compared to the use of the triangular temperature modulation. In contrast, no noticeable pyroelectric current response was detected when a spin-coated PAN film with a thickness of 10 nm was investigated (Figure 2C). The observation of a pyroelectric current in the PAN brush is remarkable as the sample was not subjected to electric field poling, and points toward a spontaneous net polar alignment of the nitrile groups in the polymer brush film. In a previous paper,^41^ we have found that surface-initiated atom transfer radical polymerization of 2-hydroxyethyl methacrylate and tert-butyl methacrylate generates brush films that do not show a pyroelectric response. This supports the notion that the pyroelectric behavior of the PAN brush is due to the dipolar nitrile side chain functional groups, and suggests the absence of any putative contributions of bromine chain end functional groups of the ATRP grown polymer grafts. The pyroelectric behavior of the PAN brush, and the absence thereof when investigating the spin-coated PAN film (Figure 2C) highlights the importance of polymer conformation. Whereas the polymer chains in the PAN brush film are aligned, and tethered with one chain end to the underlying substrate, the spin-coated PAN film lacks such macromolecular orientational order.

To assess the pyroelectric coefficient of the PAN brush, a series of measurements was carried out using temperature amplitudes varying from 1.2 to 3.1 K at a frequency of 20 mHz (Supporting Information Figure S5). Figure 2D plots the pyroelectric current density measured in these experiments as a function of Δ*T*/Δ*t*, i.e., the rate of temperature change in the experiment, not only for the PAN brush, but also, as a reference, for a commercially available, electrically poled PVDF film (thickness = 50 μm). Analysis of the slope of the line that connects the data points in Figure 2D gives the pyroelectric coefficient. From this analysis, a pyroelectric coefficient of 5.0 × 10^–5^ C/m^2^·K was obtained for the PAN brush, and 5.6 × 10^–5^ C/m^2^·K for the poled PVDF film. The pyroelectric coefficient of the PAN brush is remarkable, as it exceeds the value of ∼3.9 × 10^–5^ C/m^2^·K that has been reported for electrically poled PAN films,^44^ and is comparable to that of the electrically poled PVDF film sample. In contrast to the previously published results on solvent cast PAN films as well as the PVDF film that is also included in Figure 2D, the PAN brush displays spontaneous pyroelectricity without the need for poling in an electric field. The pyroelectric current measured on the PAN brushes did not change significantly as a function of time, not even after long thermal cycling times, as illustrated by the measurements shown in Figure 2E, which were recorded over a period of 8000 s, or after storage of the sample at room temperature for 9 months (Supporting Information Figure S6).

The results of the pyroelectric measurements indicate that surface-initiated polymerization allows to produce polyacrylonitrile brushes that display pyroelectric behavior without the need for e.g., electric field poling. In particular the absence of any noticeable pyroelectric current in the spin-coated PAN films highlights the impact of surface-initiated polymerization and polymer brush architecture on these properties. Since materials that possess pyroelectric properties must also have piezoelectric properties, piezoresponse force microscopy (PFM) experiments were done to corroborate the findings reported in Figure 2. Figure 3A shows the inverse piezoelectric response of a PAN brush and a spin-coated PAN film (both with film thicknesses of 10 nm) measured by PFM using a 3 V excitation voltage. Whereas a significant inverse piezoelectric amplitude is observed for the PAN brush, the response of the spin-coated PAN film is much smaller, which is in agreement with the results of the pyroelectric analyses presented in Figure 2. To estimate the inverse piezoelectric coefficient (*d*_33_), PFM experiments were performed using different excitation voltages. Figure 3B presents the measured piezoelectric amplitude as a function of the excitation voltage for a PAN brush with a thickness of 10 nm, as well as a spin-coated PAN film with a thickness of 10 nm. Figure 3B also includes results of PFM analyses that were performed on a commercially available, electrically poled PVDF film (thickness = 50 μm). From these experiments *d*_33_ values of 1.25 ± 0.75 pm/V, 0.625 ± 0.45 pm/V and 18.75 ± 0.15 pm/V were determined for the PAN brush, the spin coated PAN film, and the poled PVDF sample. The *d*_33_ value of the PAN brush is comparable to that reported for electrospun PAN fibers.^49^ Supporting Information Figure S7 provides 0.5 × 0.5 μm^2^ PFM images of the PAN brush, highlighting the polarization orientation across the brush sample. These results confirm the pyroelectric analyses presented earlier, and highlight that surface-initiated atom transfer radical polymerization of acrylonitrile results in the formation of polar PAN brushes that display spontaneous pyro- and piezoelectricity. Comparison of the results obtained on the PAN brush and the spin coated PAN film reiterates the impact of polymer chain conformation.

**Figure 3:**
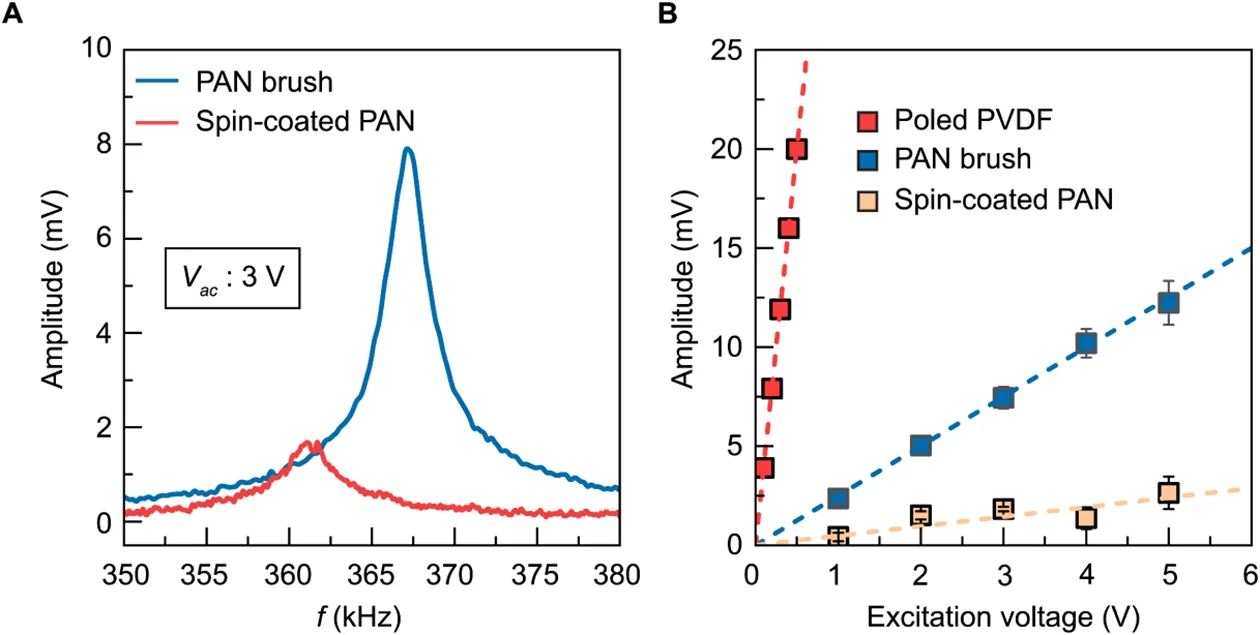
(**A**) Amplitude-excitation curves from PFM analysis of a PAN brush and a spin-coated PAN film. (**B**) Piezoelectric response of the spin-coated PAN film, the PVDF control sample, and a PAN brush versus excitation *V*_AC_.

The spontaneous pyro- and piezoelectricity observed in the PAN brushes, and the noticeable absence thereof in the spin-coated PAN film suggests that surface-initiated atom transfer radical polymerization of acrylonitrile results in a net parallel alignment of nitrile groups. To elucidate the orientation of the nitrile groups, the PAN brushes and spin coated PAN films were analyzed with polarized FTIR spectroscopy, and Near Edge X-ray Absorption Fine Structure (NEXAFS) spectroscopy experiments.

FTIR spectra of a PAN brush and a spin-coated PAN film recorded at polarization angles of 0° (∥) and 90° (⊥) are presented in Figure 4A and Figure 4B. Most prominent in these spectra are the CH_2_/CH stretch band at ∼2921 cm^–1^ and the CN stretch vibration at ∼2242 cm^–1^.^48,50^ The spectra presented in Figure 4 are normalized with respect to the intensity of the CH_2_/CH stretch vibration at ∼2921 cm^–1^. To highlight a possible polarization angle dependence of specific infrared vibrations, Figure 4C shows the corresponding differential spectra that are obtained by subtracting the spectra recorded at the different angles. Comparison of the FTIR spectra of the PAN brush at polarization angles of 0° and 90° reveals significant differences in the intensity of the CN signal at 2242 cm^–1^. A variation in intensity of the CN band is also observed for the spin-coated PAN film, however, the difference is smaller than that for the PAN brush. The significant polarization angle-dependence of the CN band intensity in the FTIR spectra of the PAN brush is indicative of a nonisotropic orientation of the nitrile groups, which is more pronounced in the polymer brush, as compared to the spin-coated PAN film.

**Figure 4:**
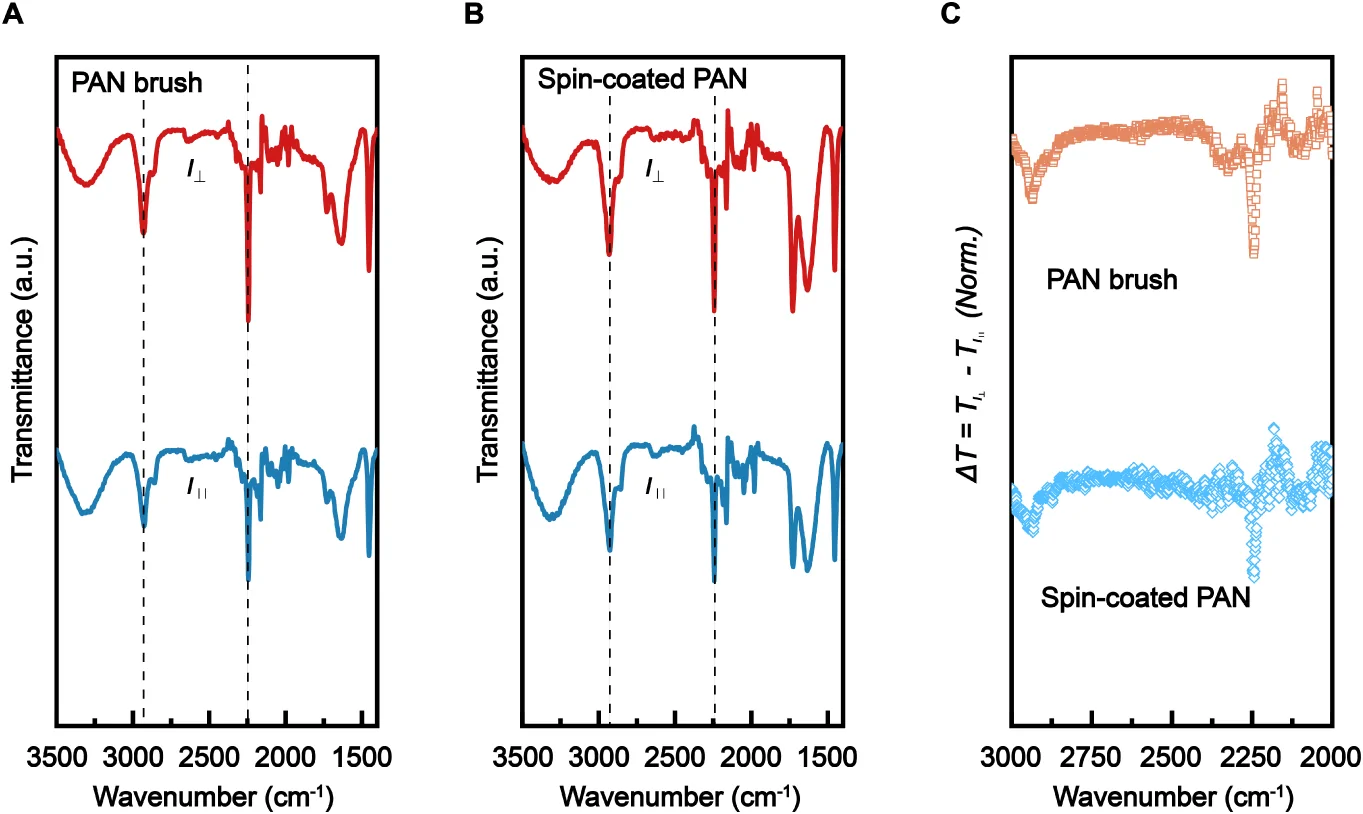
Polarized FTIR spectra of (**A**) a PAN brush, (**B**) a spin coated PAN film, and (**C**) differential transmittance FTIR spectra of a PAN brush and a spin coated PAN film.

Figure 5 presents the results of near edge X-ray absorption fine structure (NEXAFS) experiments that were performed on a PAN brush and a spin-coated PAN film at different angles of polarization. The C K edge spectra in Figure 5A feature π*_C≡N_ resonances at 286 and 288 eV, and the σ*_C–H_ resonance at 293 eV.^51,52^ The splitting of the π levels in these spectra is ascribed to intramolecular CN interactions. The full C K-edge NEXAFS spectra of the PAN brush are included in Supporting Information Figure S8. Shown in Figure 5A are NEXAFS spectra that were obtained using polarized X-rays at polarization angles of 0° and 60°. Figure 5B plots the variation of the intensity of the π*_C≡N_ resonance at 288 eV as a function of the angle of polarization of the incident X-ray beam. Whereas for the spin-coated PAN film only a small polarization angle-dependence of the π*_C≡N_ resonance intensity was observed, analysis of the PAN brush reveals a more pronounced dependence of the π*_C≡N_ resonance intensity on the angle of polarization of the incident X-rays. These results corroborate the insights obtained from the polarized FTIR experiments, and suggest an overall nonisotropic orientation of CN groups in the PAN brush, whereas the CN groups in the spin coated PAN film are more isotropically oriented. The nonisotropic orientation of CN groups in the polymer brush is consistent with the spontaneous pyro- and piezoelectricity that is observed for the PAN brushes. The C K-edge absorption NEXAFS spectrum of the PAN brush features two well-defined π*_C≡N_ transitions, whereas these are less clearly featured in the spectrum from the spin coated PAN film, which may reflect differences in tacticity.^49,50^ While the commercially available spin coated PAN is largely atactic, the well-defined π*_C≡N_ transitions in the spectrum of the PAN brush suggest a higher degree of isotacticity.^49,53^ As an isotactic placement of acrylonitrile repeat units enforces alignment of the nitrile groups along the polymer backbone, this could facilitate and contribute to the nonisotropic orientation of these dipolar groups in the polymer brush.

**Figure 5:**
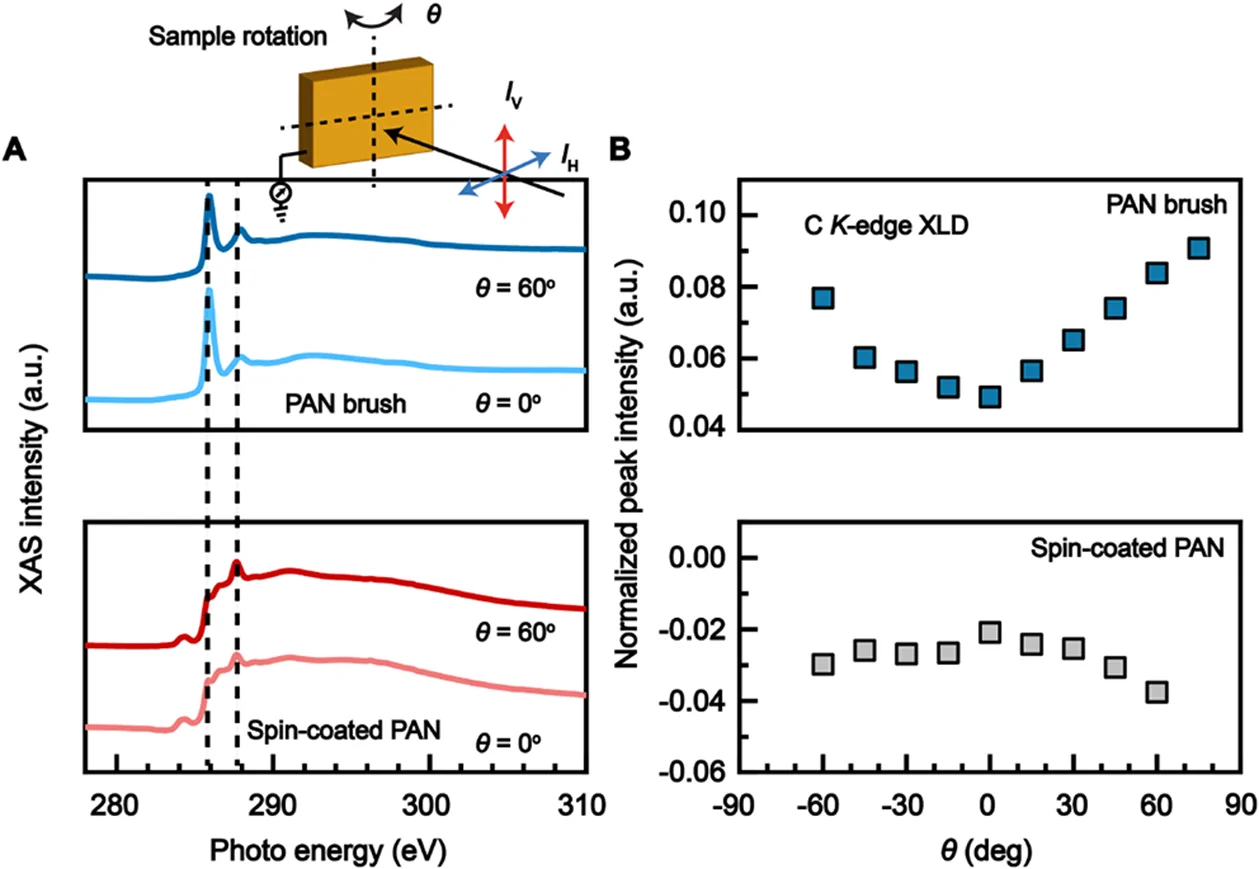
(**A**) C K-edge absorption spectra of the PAN brush and the spin coated PAN film measured at angles of polarization of 0° and 60°. (**B**) variation of the intensity of the π*_C≡N_ resonance at 288 eV as a function of the angle of polarization of the incident X-ray beam for the PAN brush and the spin coated PAN film.

## Conclusions

The results presented in this paper indicate that surface-initiated atom transfer radical polymerization of acrylonitrile, a vinyl monomer with a dipolar nitrile side chain functional group, results in the formation of thin polymer brush films that display spontaneous pyro- and piezoelectric behavior. As these properties are not observed in spin-coated PAN films, this suggests that surface-initiated growth of PAN enforces a net parallel orientation of the dipolar nitrile groups. This is supported by the results of polarized FTIR and NEXAFS experiments, which suggest an overall nonisotropic orientation of CN groups in the PAN brush, whereas the CN groups in the spin coated PAN film are more isotropically oriented. The ability to produce thin polymer films that possess spontaneous polar order is attractive as it obviates the need for electrical field poling or other postprocessing steps that are generally applied to augment the pyro- and piezoelectric properties of polymer materials.

## Supplementary Material



## Data Availability

The source data of this study are available from the Zenodo repository at DOI: https://doi.org/10.5281/zenodo.22117056.

## Notes

Characterization data of the PAN brushes; additional dynamic pyroelectric current measurements (PDF).

## References

[1] Wada Y., Hayakawa R. (1976). Piezoelectricity
and Pyroelectricity of Polymers. Jpn. J. Appl.
Phys.

[2] Lang S. B., Muensit S. (2006). Review of Some Lesser-Known Applications of Piezoelectric
and Pyroelectric Polymers. Appl. Phys. A: mater.
Sci. Process.

[3] Park C., Lee K., Koo M., Park C. (2021). Soft Ferroelectrics
Enabling High-Performance
Intelligent Photo Electronics. Adv. Mater.

[4] Wu Y., Ma Y., Zheng H., Ramakrishna S. (2021). Piezoelectric Materials for Flexible
and Wearable Electronics: A Review. Mater. Des.

[5] Zhang D., Wu H., Bowen C. R., Yang Y. (2021). Recent Advances in Pyroelectric Materials
and Applications. Small.

[6] Chen G., Xiao X., Zhao X., Tat T., Bick M., Chen J. (2022). Electronic Textiles for Wearable
Point-of-Care Systems. Chem. Rev.

[7] Lay R., Deijs G. S., Malmström J. (2021). The Intrinsic Piezoelectric Properties
of Materials – a Review with a Focus on Biological Materials. RSC Adv.

[8] Kim D., Han S. A., Kim J. H., Lee J.-H., Kim S.-W., Lee S.-W. (2020). Biomolecular Piezoelectric
Materials: From Amino Acids
to Living Tissues. Adv. Mater.

[9] Kepler R. G., Anderson R. A. (1992). Ferroelectric Polymers. Adv.
Phys.

[10] Usher T. D., Cousins K. R., Zhang R., Ducharme S. (2018). The Promise
of Piezoelectric
Polymers. Polym. Int.

[11] Mishra S., Unnikrishnan L., Nayak S. K., Mohanty S. (2019). Advances in Piezoelectric
Polymer Composites for Energy Harvesting Applications: A Systematic
Review. Macromol. Mater. Eng.

[12] Lovinger A. J. (1983). Ferroelectric
Polymers. Science.

[13] Kepler, R. G. F. Pyroelectric, and Piezoelectric Properties of Poly­(Vinylidene Fluoride). Ferroelectric Polymers: chemistry: physics, and Applications. Nalwa, H. S. pp. 183–232.CRC Press, 1995

[14] Ameduri B. (2009). From Vinylidene
Fluoride (VDF) to the Applications of VDF-Containing Polymers and
Copolymers: Recent Developments and Future Trends. Chem. Rev.

[15] Martins P., Lopes A. C., Lanceros-Mendez S. (2014). Electroactive Phases of Poly­(Vinylidene
Fluoride): Determination, Processing and Applications. Prog. Polym. Sci.

[16] Zapsas G., Patil Y., Gnanou Y., Ameduri B., Hadjichristidis N. (2020). Poly­(Vinylidene
Fluoride)-Based Complex Macromolecular Architectures: From Synthesis
to Properties and Applications. Prog. Polym.
Sci.

[17] Tasaka, S. Cyanopolymers. In Ferroelectric Polymers: chemistry: physics, and Applications, Nalwa, H. S. , Eds.; CRC Press, 1995, Vol. 7, pp. 325–352

[18] Nalwa, H. S. Ferroelectric Nylons. Ferroelectric Polymers: chemistry: physics, and Applications. Nalwa, H. S. pp. 281–323.CRC Press, 1995

[19] Halasyamani P. S., Poeppelmeier K. R. (1998). Noncentrosymmetric
Oxides. Chem.
Mater.

[20] Wachtel E., Lubomirsky I. (2010). Quasi-Amorphous Inorganic Thin Films: Non-Crystalline
Polar Phases. Adv. Mater.

[21] Zhao B., Brittain W. J. (2000). Polymer brushes:
surface-immobilized macromolecules. Prog. Polym.
Sci.

[22] Pyun J., Kowalewski T., Matyjaszewski K. (2003). Synthesis of polymer brushes using
atom transfer radical polymerization. Macromol.
Rapid Commun.

[23] Edmondson S., Osborne V. L., Huck W. T. S. (2004). Polymer
brushes via surface-initiated
polymerizations. Chem. Soc. Rev.

[24] Barbey R., Lavanant L., Paripovic D., Schüwer N., Sugnaux C., Tugulu S., Klok H.-A. (2009). Polymer Brushes
via Surface-Initiated Controlled Radical Polymerization: Synthesis,
Characterization, Properties, and Applications. Chem. Rev.

[25] Zoppe J., Cavusoglu Ataman N., Mocny P., Wang J., Moraes J., Klok H.-A. (2017). Surface-Initiated
Controlled Radical Polymerization:
State-of-the-Art, Opportunities, and Challenges in Surface and Interface
Engineering with Polymer Brushes. Chem. Rev.

[26] Pester C. W., Klok H.-A., Benetti E. M. (2023). Opportunities,
Challenges, and Pitfalls
in Making, Characterizing, and Understanding Polymer Brushes. Macromolecules.

[27] Deckers S., Bloemen M., Koeckelberghs G., Glorieux C., Verbiest T., van der Veen M. A. (2017). Conformational
changes of a surface-tethered polymer
during radical growth probed with second-harmonic generation. Langmuir.

[28] Uekusa T., Nagano S., Seki T. (2007). Unique molecular
orientation in a
smectic liquid crystalline polymer film attained by surface-initiated
graft polymerization. Langmuir.

[29] Uekusa T., Nagano S., Seki T. (2009). Highly Ordered In-Plane Photoalignment
Attained by the Brush Architecture of Liquid Crystalline Azobenzene
Polymer. Macromolecules.

[30] Haque H. A., Hara M., Nagano S., Seki T. (2013). Photoinduced In-Plane
Motions of Azobenzene Mesogens Affected by the Flexibility of Underlying
Amorphous Chains. Macromolecules.

[31] Peng B., Rühe J., Johannsmann D. (2000). Homogenously aligned liquid-crystal
polymer brushes. Adv. Mater..

[32] Hamelinck P. J., Huck W. T. S. (2005). Homeotropic alignment
on surface-initiated liquid crystalline
polymer brushes. J. Mater. Chem.

[33] Li X., Yanagimachi T., Bishop C., Smith C., Dolejsi M., Xie H., Kurihara K., Nealey P. F. (2018). Engineering the anchoring behavior
of nematic liquid crystals on a solid surface by varying the density
of liquid crystalline polymer brushes. Soft
Matter.

[34] Schüwer N., Klok H.-A. (2010). A Potassium-Selective
Quartz Crystal Microbalance Sensor
Based on Crown-Ether Functionalized Polymer Brushes. Adv. Mater.

[35] Hiller W., Grabe B. (2023). The Universal Calibration
for Structure- and Solvent-Independent
Molar Mass Determinations of Polymers Using Diffusion-Ordered Spectroscopy. Anal. Chem.

[36] Refractive Index of Polymers by Index – scipoly.com. https://scipoly.com/technical-library/refractive-index-of-polymers-by-index/ (accessed 01.07.2026).

[37] Barla A., Nicolás J., Cocco D., Valvidares S., Herrero-Martín J., Gargiani P., Moldes J., Ruget C., Pellegrin E., Ferrer S. (2016). Design and Performance
of BOREAS, the Beamline for Resonant X-Ray Absorption and Scattering
Experiments at the ALBA Synchrotron Light Source. J. Synchrotron Radiat.

[38] Daglish M. (1998). A Dynamic
Method for Determining the Pyroelectric Response of Thin Films. Integr. Ferroelectr.

[39] Davis M., Damjanovic D., Setter N. (2004). Pyroelectric Properties of (1–x)­Pb­(Mg1/3Nb2/3)­O3-xPbTiO3
and (1–x)­Pb­(Zn1/3Nb2/3)­O3-xPbTiO3 Single Crystals Measured
Using a Dynamic Method. J. Appl. Phys.

[40] Lubomirsky I., Stafsudd O. (2012). Invited Review
Article: Practical Guide for Pyroelectric
Measurementsa). Rev. Sci. Instrum.

[41] Wang J., Hu F., Sant S., Chu K., Riemer L., Damjanovic D., KilbeyIi S. M., Klok H.-A. (2024). Pyroelectric
Polyelectrolyte Brushes. Adv. Mater.

[42] Ueda H., Carr S. H. (1984). Piezoelectricity in Polyacrylonitrile. Polym. J.

[43] Likhovidov V. S., Golovanov V. V., Vannikov A. V. (1978). Pyroelectric Properties and the Nature
of the Polarization of Polyacrylonitrile. Polym.
Sci. U.S.S.R.

[44] Stephens A. W., Levine A. W., Fech J., Zrebiec T. J., Cafiero A. V., Garofalo A. M. (1974). Pyroelectric Polymer Films. Thin
Solid Films.

[45] von
Berlepsch H., Künstler W. (1988). Piezoelectricity in Acrylonitrile/Methylacrylate
Copolymer. Polym. Bull.

[46] Pearson R. M., Lim C.-H., McCarthy B. G., Musgrave C. B., Miyake G. M. (2016). Organocatalyzed
Atom Transfer Radical Polymerization Using N-Aryl Phenoxazines as
Photoredox Catalysts. J. Am. Chem. Soc.

[47] Li L., Chan C.-M., Weng L. T. (1997). Effects of the Sequence Distribution
of Poly­(Acrylonitrile–butadiene) Copolymers on the Surface
Chemical Composition As Determined by XPS and Dynamic Contact Angle
Measurements. Macromolecules.

[48] Tas S., Kaynan O., Ozden-Yenigun E., Nijmeijer K. (2016). Polyacrylonitrile
(PAN)/Crown Ether Composite Nanofibers for the Selective Adsorption
of Cations. RSC Adv.

[49] Yu S., Milam-Guerrero J., Tai Y., Yang S., Choi Y. Y., Nam J., Myung N. V. (2022). Maximizing
Polyacrylonitrile Nanofiber Piezoelectric
Properties through the Optimization of Electrospinning and Post-Thermal
Treatment Processes. ACS Appl. Polym. Mater.

[50] Chen M., Wang C., Fang W., Wang J., Zhang W., Jin G., Diao G. (2013). Electrospinning of Calixarene-Functionalized Polyacrylonitrile
Nanofiber Membranes and Application as an Adsorbent and Catalyst Support. Langmuir.

[51] Tourillon G., Garrett R., Lazarz N., Raynaud M., Reynaud C., Lécayon G., Viel P. (1990). A NEXAFS Study of Thin Polyacrylonitrile
Films Electrochemically Deposited on Ni: The Effect of the Film Thickness
and Annealing Treatment. J. Electrochem. Soc.

[52] Lecayon G., Didier I., Viel P., Defranceschi M., Tourillon G., Delhalle J. (1993). Preliminary NEXAFS Study of Polyacrylonitrile
Samples Differing by Their Isotacticity Percentages. J. Phys. Chem.

[53] Street R. M., Minagawa M., Vengrenyuk A., Schauer C. L. (2019). Piezoelectric Electrospun
Polyacrylonitrile with Various Tacticities. J. Appl. Polym. Sci.

