# Supplementary material for: Spontaneously Piezo- and Pyroelectric Polyacrylonitrile Brushes Prepared via Surface-Initiated Polymerization
Source: J Am Chem Soc. 2026 Aug 28;148(35):38196–204. doi: 10.1021/jacs.6c10834 (PMC13564435; doi:10.1021/jacs.6c10834)
Supplement: Supplementary file 1 [file ja6c10834_si_001.pdf]

Supporting Information

Spontaneously Piezo- and Pyroelectric Polyacrylonitrile Brushes Prepared via Surface-Initiated Polymerization

Fei Hu[1,±], Dae-Sung Park[2,±], Suqiu Jiang,[1] Javier Herrero-Martin,[3] Konstantin Romanyuk,[4] Andrei Kholkin,[4,5] Dragan Damjanovic[2] and Harm-Anton Klok[1]*

1. Institut des Matériaux and Institut des Sciences et Ingénierie Chimiques, Laboratoire des Polymères, École Polytechnique Fédérale de Lausanne (EPFL), Station 12, CH-1015 Lausanne, Switzerland.
2. Group for Ferroelectrics and Functional Oxides, École Polytechnique Fédérale de Lausanne (EPFL), Station 12, CH-1015 Lausanne, Switzerland.
3. ALBA Synchrotron, Carrer de la Llum 2-26, Cerdanyola del Vallès 08290, Spain
4. Department of Physics, CICECO – Aveiro Institute of Materials, University of Aveiro, 3810-193, Aveiro, Portugal.
5. Institute of Solid State Physics, University of Latvia, LV-1063, Riga, Latvia.

Author email addresses: fei.hu@epfl.ch, dspark1980@gmail.com, suqiu.jiang@epfl.ch, jherrero@cells.es, k.romanyuk@ua.pt, andrei.kholkin@cfi.lu.lv, dragan.damjanovic@epfl.ch, harm-anton.klok@epfl.ch

± These authors contributed equally. * Corresponding author

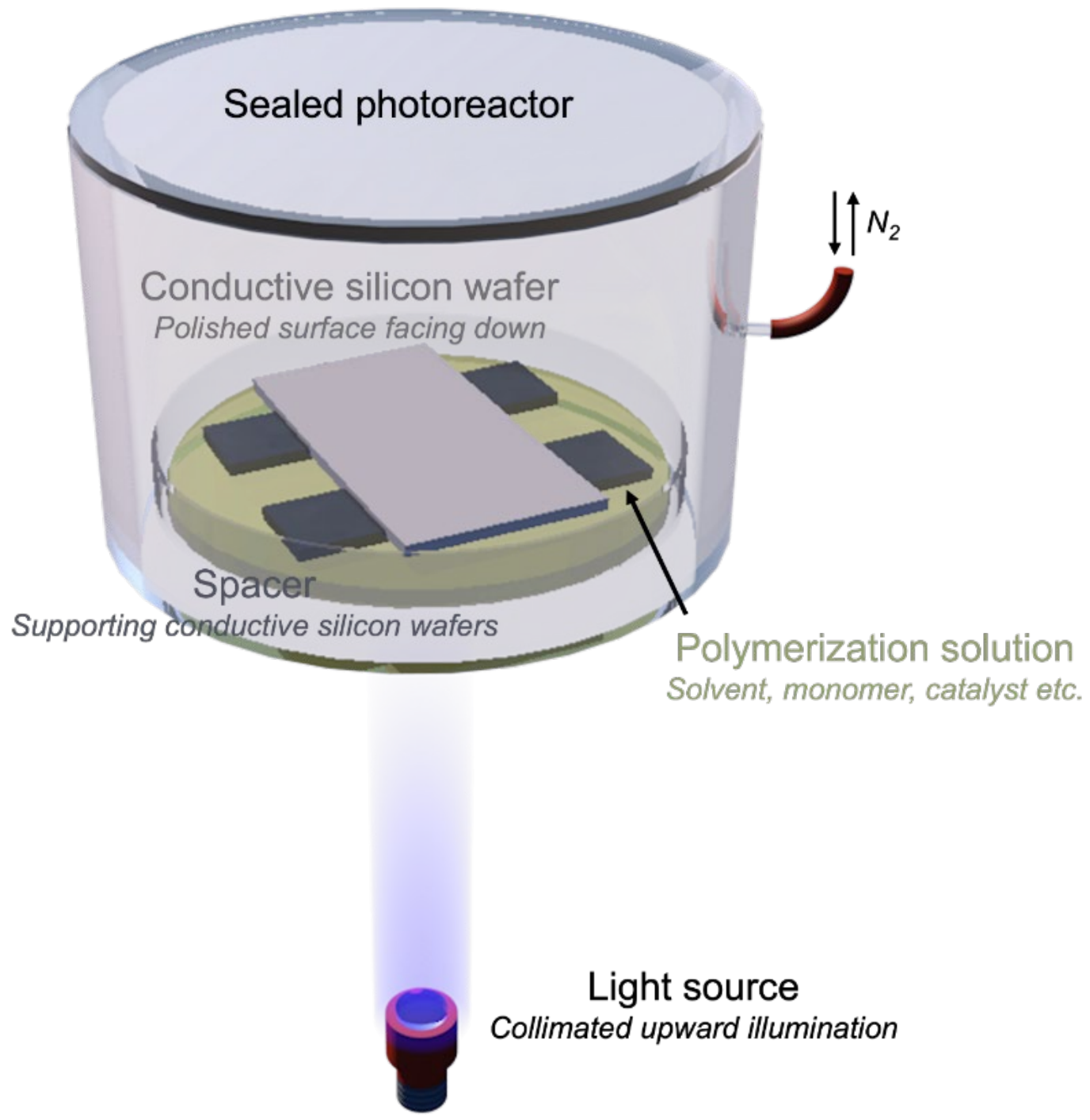

Figure S1. Schematic illustration of the set-up that was used for the photo-induced SI-ATRP of acrylonitrile.

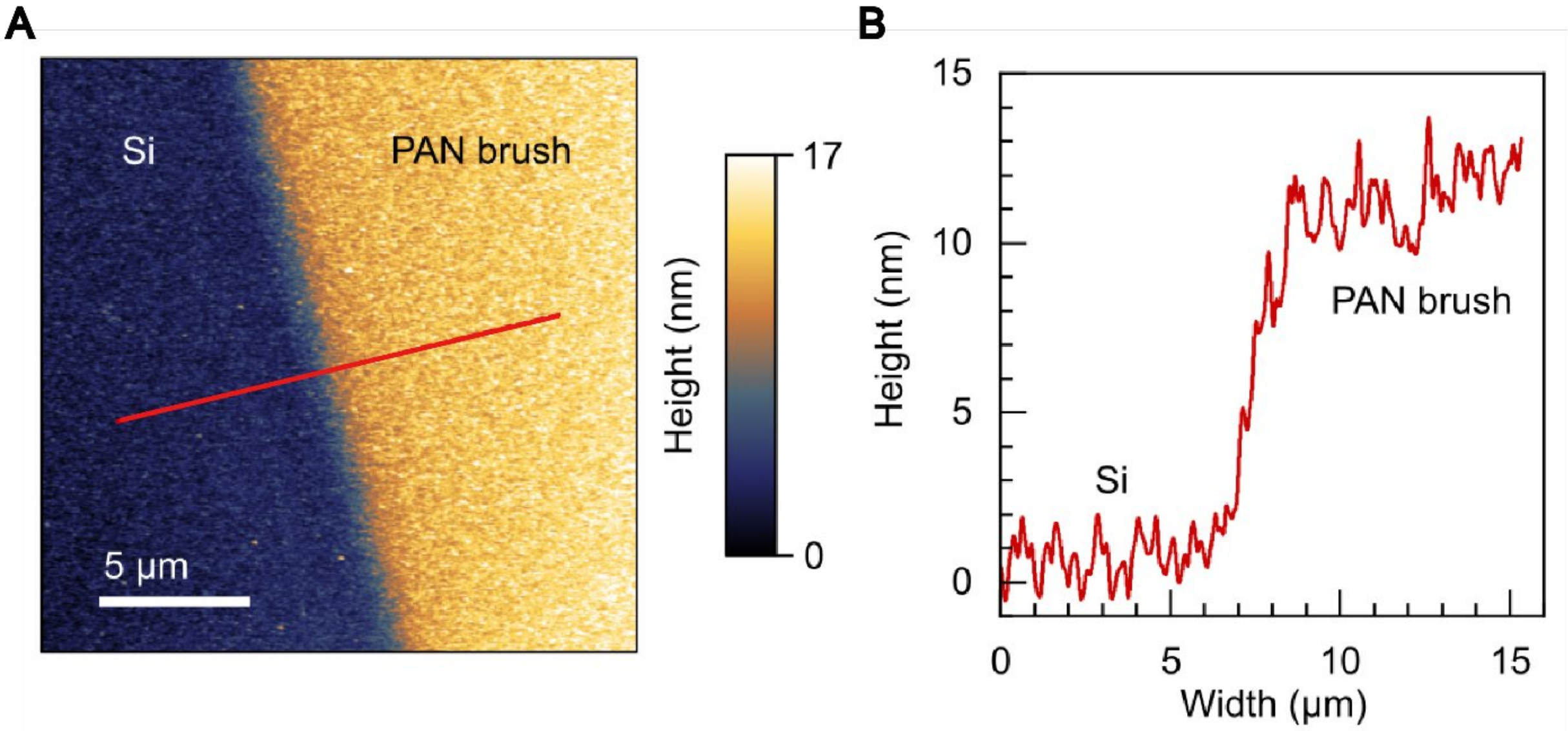

Figure S2. (A) AFM image, and (B) cross-sectional profile recorded at the edge of a PAN brush that was prepared with a polymerization time of 4 hrs.

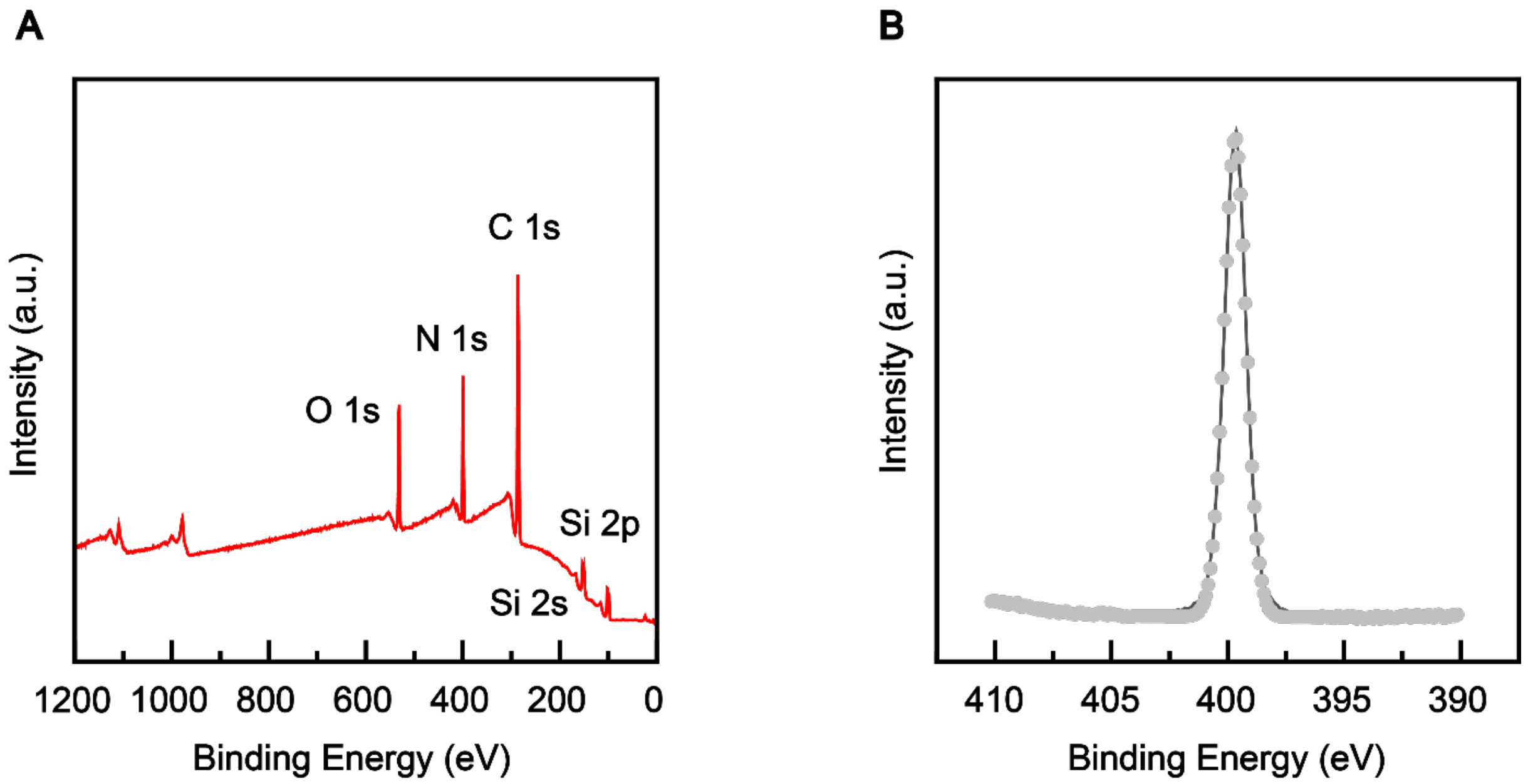

Figure S3: (A) Survey XPS spectrum of the PAN brush; (B) N1s high resolution scan of the PAN brush.

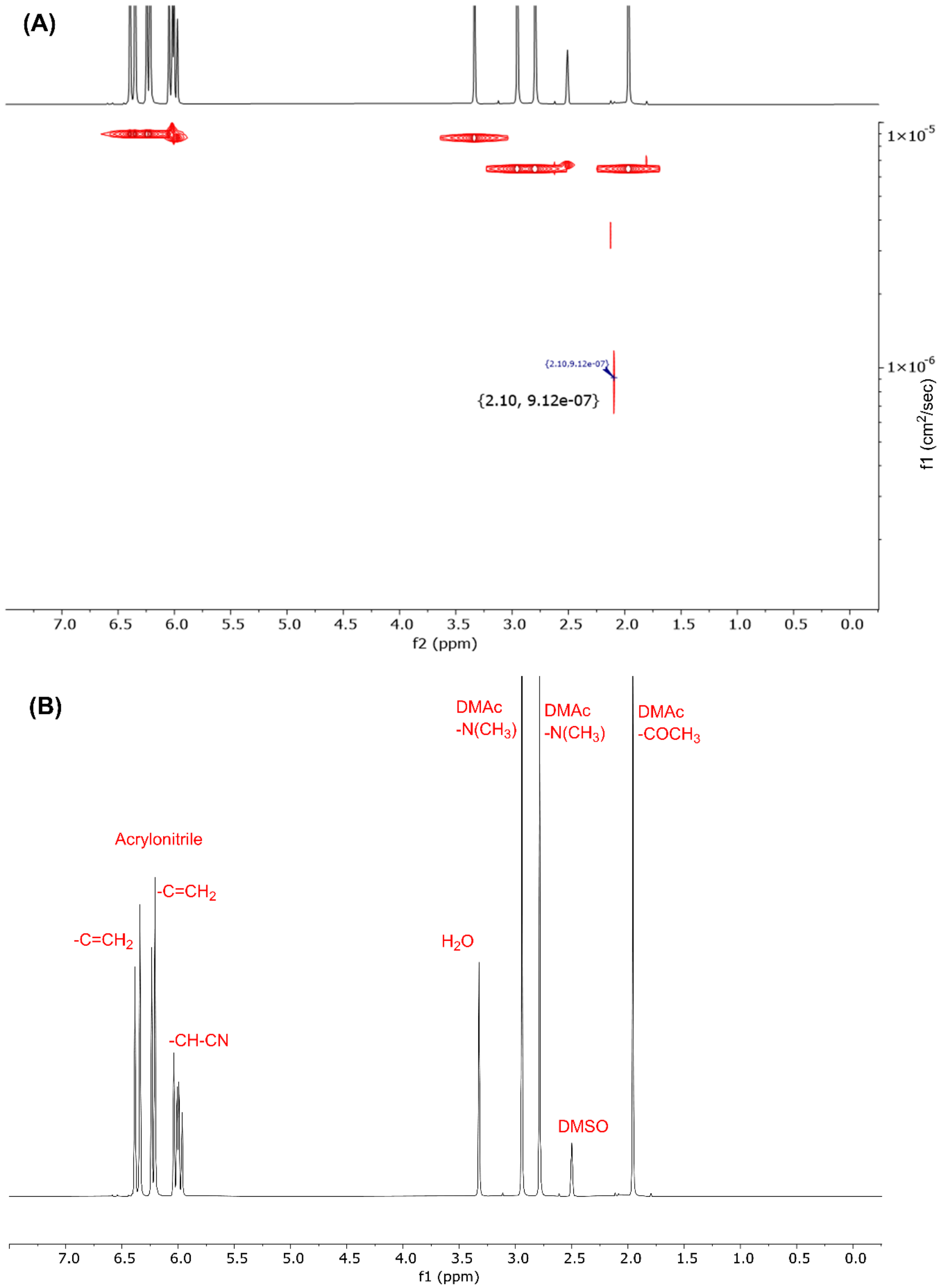

Figure S4: (A) DOSY ^{1}H NMR spectrum, and (B) ^{1}H-NMR spectrum of the DMAc solution after surface-initiated atom transfer radical polymerization of acrylonitrile in the presence of a sacrificial initiator (spectra were recorded in DMSO-d_6 at 298 K).

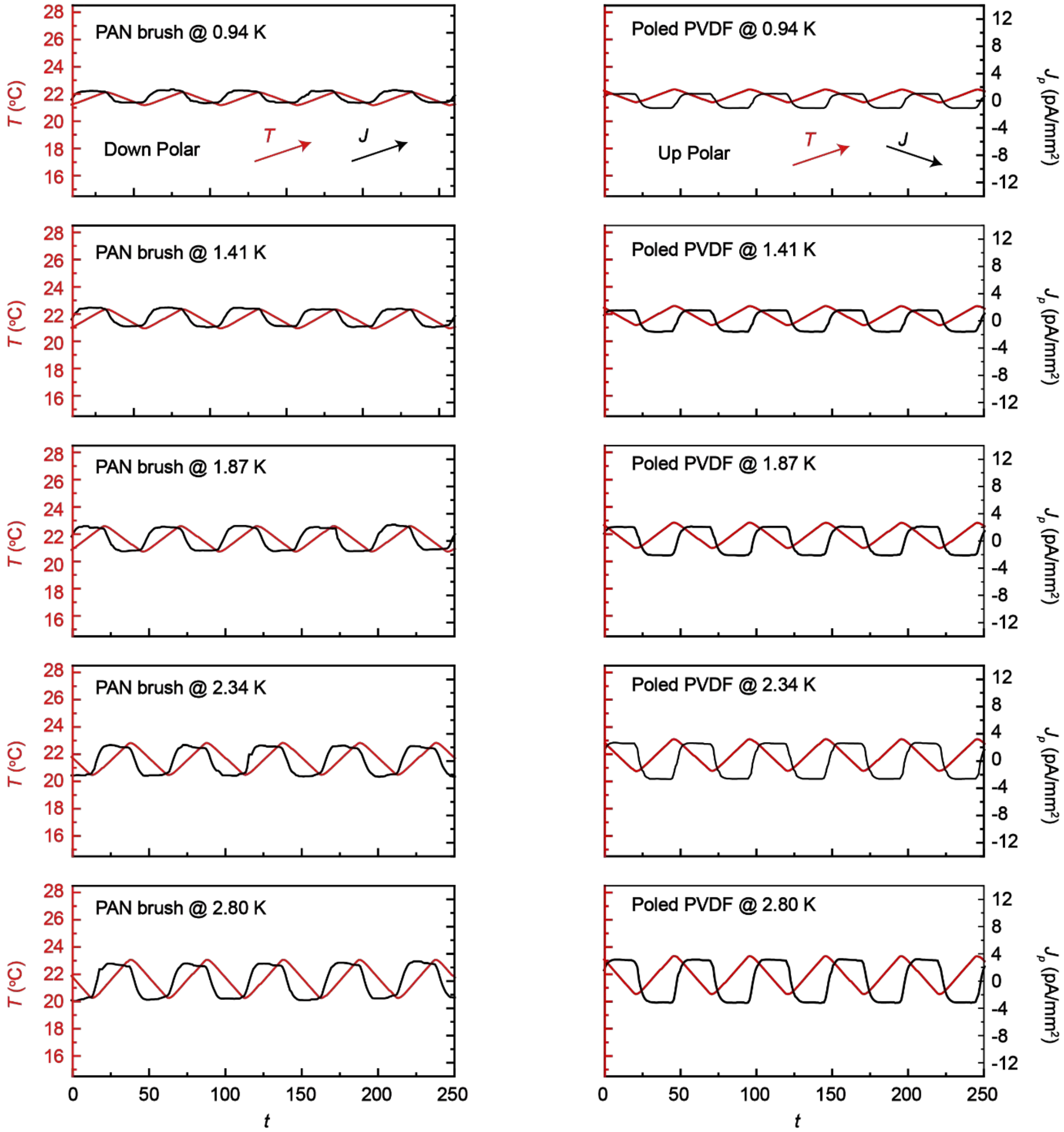

Figure S5. The pyroelectric current density, J_p, of a PAN brush (left) and a commercially available PVDF film (right) measured by continuously applying triangular temperature waveforms in time (t) at different temperature amplitudes (Δ T) (from 0.94 to 2.80 K) at a frequency of 20 mHz.

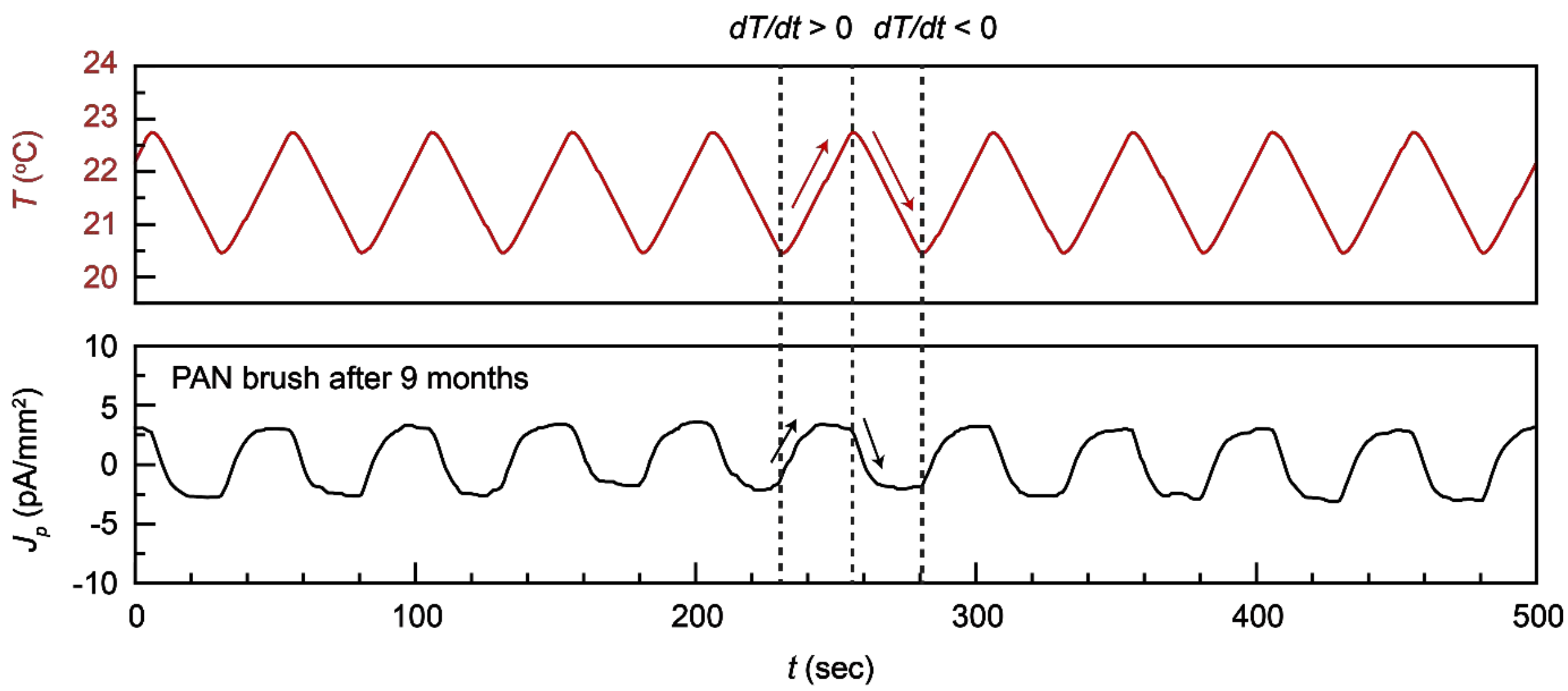

Figure S6. The pyroelectric current density, J_{p}, of a PAN brush (ΔT = 2.4 K and f = 20 mHz) after storage at room temperature for 9 months.

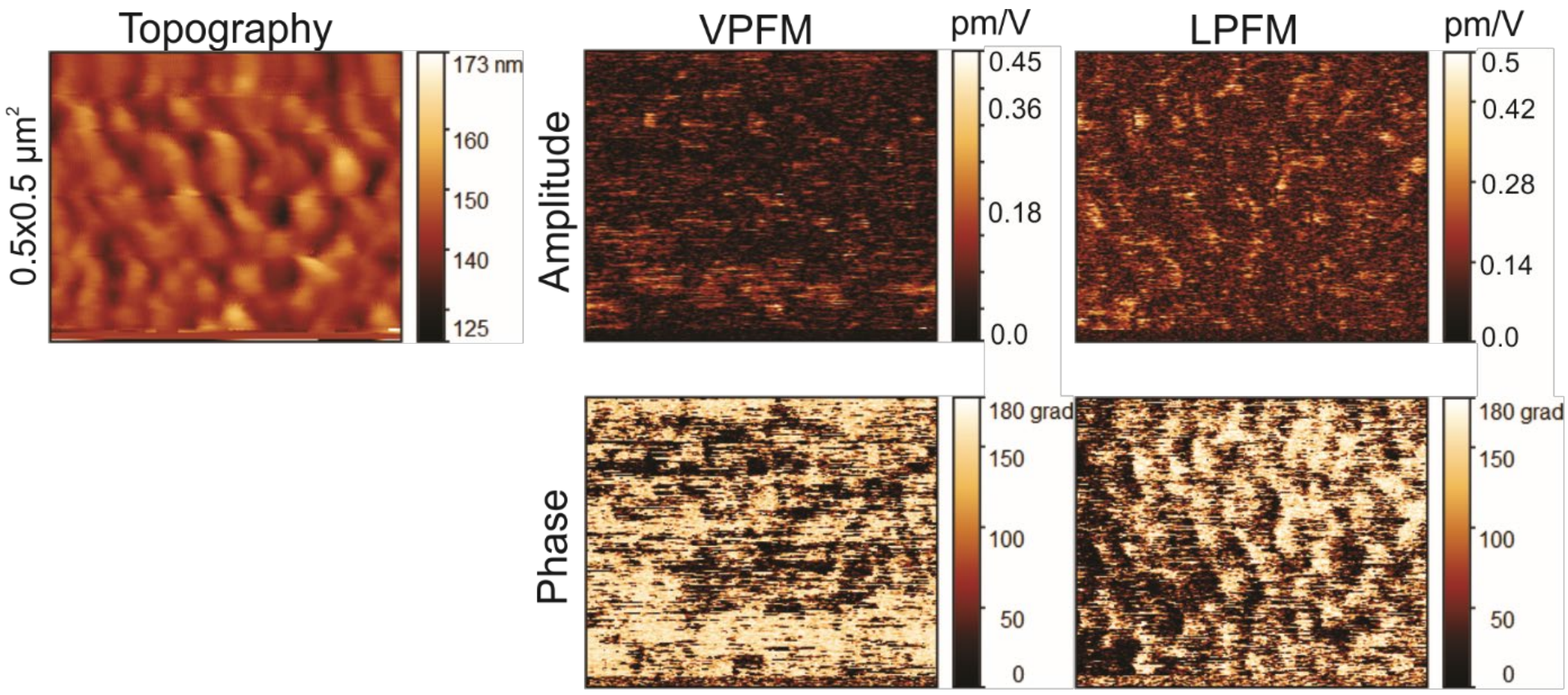

Figure S7. The topography, vertical piezoresponse force microscopy (VPFM), lateral piezoresponse force microscopy (LPFM), amplitude and phase images measured for the PAN brush.

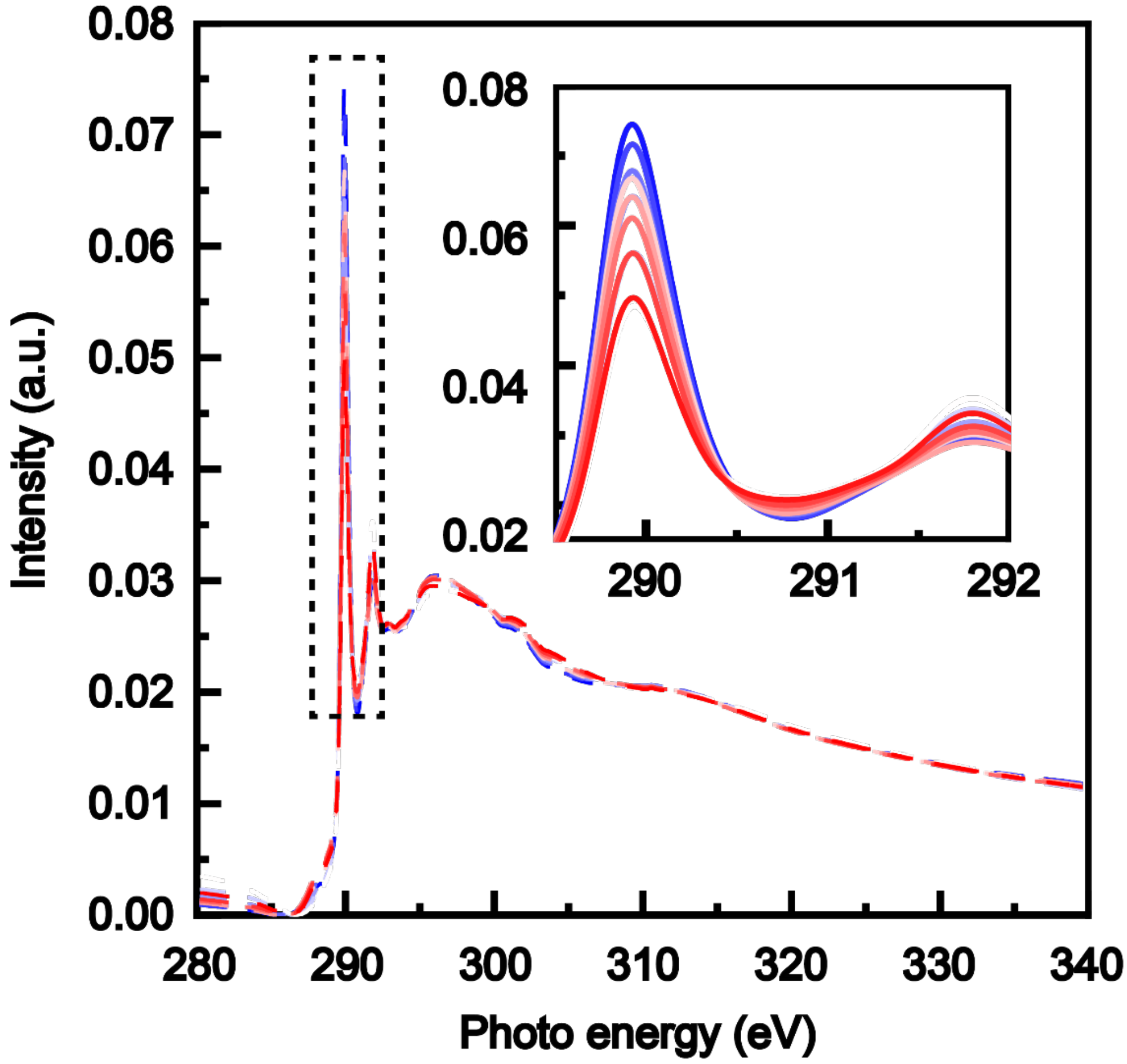

Figure S8. C K-edge XAS spectra of a PAN brush measured at different X-ray incident angles showing the systematic variation of the CN π^* feature with θ.
